# Phase transitions of ordered ice in graphene nanocapillaries and carbon nanotubes

**DOI:** 10.1038/s41598-018-22201-3

**Published:** 2018-03-01

**Authors:** Muralikrishna Raju, Adri van Duin, Matthias Ihme

**Affiliations:** 1 Department of Mechanical Engineering, Stanford University, Stanford, CA 94305 USA; 2 Department of Mechanical and Nuclear Engineering, The Pennsylvania State University, University Park, PA 16802 USA

## Abstract

New phase diagrams for water confined in graphene nanocapillaries and single-walled carbon nanotubes (CNTs) are proposed, identifying ice structures, their melting points and revealing the presence of a solid-liquid critical point. For quasi-2D water in nanocapillaries, we show through molecular-dynamics simulations that AA stacking in multilayer quasi-2D ice arises from interlayer hydrogen-bonding and is stable up to three layers, thereby explaining recent experimental observations. Detailed structural and energetic analyses show that quasi-2D water can freeze discontinuously through a first-order phase transition or continuously with a critical point. The first-order transition line extends to a continuous transition line, defined by a sharp transition in diffusivity between solid-like and liquid-like regimes. For quasi-1D water, confined in CNTs, we observe the existence of a similar critical point at intermediate densities. In addition, an end point is identified on the continuous-transition line, above which the solid and liquid phases deform continuously. The solid-liquid phase transition temperatures in CNTs are shown to be substantially higher than 273 K, confirming recent Raman spectroscopy measurements. We observe ultrafast proton and hydroxyl transport in quasi-1D and -2D ice at 300 K, exceeding those of bulk water up to a factor of five, thereby providing possible applications to fuel-cells and electrolyzers.

## Introduction

Water molecules confined in nanometer-sized pores or adsorbed at interfaces exhibit unusual phase behavior that deviates significantly from bulk water due to confinement effects^1–6^. The introduction of confinement and competition of fluid-wall and fluid-fluid forces can lead to surface-driven phase changes, thereby shifting the phase boundary. Low-dimensional water is ubiquitous and critical to biological processes^7^ as well as attractive for various applications in materials science, geology and nanotechnology^8–10^, including proton fuel cells^7^, nanoscale ferroelectric devices^11,12^, flow sensors^13^ and high-flux desalination membranes^14,15^. In addition, it is of fundamental scientific interest to understand the new physics resulting from finite-size effects, changing dimensionality and fluid-wall interactions. Of particular relevance is extreme nanoconfinement where water molecules are only a few layers thick and the confinement length is in the range of intermolecular forces leading one to expect large shifts in phase coexistence curves^16,17^. The Gibbs-Thomson equation based on classical continuum thermodynamics, relating the shift in freezing temperature to the average pore diameter, breaks down below a confinement length of ~2.5 nm^17,18^. A great variety of new phases has been observed experimentally and theoretically in this regime, and computational studies have reported the possibility of a solid-liquid critical point^3,19,20^. However, major gaps in the understanding of the structures and phase transitions of low-dimensional water remain. It is of interest to explore the global phase behavior of low-dimensional water in extreme confinement to help answer several important aspects of their phase behavior: for instance, the pore-size dependence of the melting point, conditions for abrupt (first-order) versus continuous melting or the existence of a critical point along the solid-liquid coexistence line.

The widely accepted view is that the solid-liquid phase transition line does not terminate at a critical point^21^. This is largely based on traditional symmetry arguments and lack of experimental observations, although the non-existence of the solid-liquid critical point has not been proven rigorously^21,22^. Theory by Kosterlitz, Thouless, Halperin, Nelson and Young (KTHNY) suggests that two-dimensional solids may melt by a sequence involving two continuous transitions^23^, one from the solid phase to the hexatic phase followed by a second continuous transition from the hexatic phase to the liquid phase. However it is still contentious whether KTHNY theory provides the only melting mechanism in two dimensions^23,24^. In recent years, the possible existence of a solid-liquid critical point has been reported by computational studies of quasi one-dimensional (1D) and two-dimensional (2D) nanoconfined systems, exhibiting discontinuous and continuous changes in thermodynamic functions or other order parameters^3,19,25,26^.

From an experimental perspective, advancements in preparing high-purity single-walled carbon nanotubes (SWCNTs) and single-layer graphene sheets enable the exciting possibility of investigating confined water in quasi-1D and -2D cavities at nano- or sub-nanometer confinement lengths. Recent transmission electron microscopy (TEM) studies report observations of quasi-2D monolayer, bilayer and trilayer square ice in graphene nanocapillaries at room temperature^1^. Additionally, quasi-1D square ‘ice nanotubes’ have been reported in neutron diffraction^27^, nuclear magnetic resonance (NMR)^28^ and vibrational spectroscopy studies^29^ of carbon nanotubes (CNTs). However, a detailed experimental exploration of the phase diagram of 1D and 2D nanoconfined water has not been achieved yet. Computational studies of nanoconfined water could provide considerable insight into the structures and phase transitions of nanoconfined water and furnish details that are not accessible experimentally. However, structures and phases of low-dimensional water reported in computational studies have been sensitive to modeling conditions and are sometimes conflicting^4,6,19,30–32^. By addressing these issues, we perform atomic-scale simulations with molecular dynamics (MD) on experimentally validated quasi-1D and -2D ice structures in graphene and SWCNTs, respectively, to investigate their phase diagrams. Our simulations predict substantial shifts in the freezing point of nanoconfined water with an appreciable sensitivity to confinement length. The predicted freezing points are in agreement with recent experimental Raman spectroscopy measurements of nanoconfined water in CNTs^2^. In addition, our results reveal the possibility that a solid-liquid phase transition in confinement can occur discontinuously or continuously, depending on the density. This results in a pseudo-critical regime at intermediate densities and a single-phase region at high densities. We observe that the ordered, denser hydrogen bond network in these ice structures enhances both proton and hydroxyl diffusion even at temperatures in excess of 300 K. In addition, we show that the confinement length scale critically determines the rate of proton and hydroxyl diffusion. The protons are shuttled via the Grotthuss^33^ relay mechanism through the formation and concurrent cleavage of covalent bonds involving neighboring water molecules.

Recent experimental studies by Algara-Siller *et al*.^1^ observe ‘square ice’ in graphene nanocapillaries at room temperature, exhibiting symmetries that differ from the conventional tetrahedral hydrogen bonding between water molecules. However, their MD studies could not reproduce the AA stacking in bilayer and trilayer ice and instead predict AB stacking for bi- and trilayer square ice; such ordered ice was seen at pressures greater than 1.0 GPa and the AB stacked structures were devoid of interlayer hydrogen bonding. Our MD simulations employing the ReaxFF reactive force field reproduce the AA stacking for multilayer ice and predict a structure in which the layers are held together by interlayer hydrogen bonding. We investigate the phase transitions in AA stacked multilayer hexagonal and square ice held together by interlayer hydrogen bonding. MD studies by Mario *et al*.^34^ report AA stacking in bilayer square ice with a 1.2 Å in-plane shift and additionally, the multilayer ice reported in their studies are devoid of interlayer hydrogen bonding.

MD studies by Koga *et al*.^3^ and Mochizuki *et al*.^20^ have predicted the exciting possibility of a solid-liquid critical point for water confined in SWCNTs. These studies investigated water confined in SWCNTs with diameters ranging from 11.1 Å to 14.2 Å. We investigate phase transitions of water confined in SWCNTs with diameters ranging from 8.1 Å to 12.1 Å. At smaller SWCNT diameters we observe trigonal ice nanotubes, not considered in previous MD studies^3,20^ and observe that thermodynamic, structural and transport properties cannot distinguish the solid and liquid-like phases during their melting phase transition. Extreme confinement can give rise to interesting behavior as recently reported in Raman spectroscopy experiments^2^. Agrawal *et al*.^2^ investigated water confined in SWCNTs with diameters ranging from 10.5 Å to 15.2 Å and report freezing transitions at temperatures as high as 138 °C for 10.5 Å SWCNTs.

We carry out MD simulations of water molecules confined between graphene sheets using the ReaxFF reactive force field method^35^. This force field method enables investigations of proton and hydroxyl diffusion in nanoconfined water. The first-principles-based ReaxFF reactive force field method^35^ is a bond-order based force field method with a polarizable charge model, which enables the method to model the breaking and formation of bonds and the associated charge rearrangements during an energy-conserving MD simulation. Here, the water-wall interactions were modeled using the C/O/H ReaxFF force field parameters employed in Hatzell *et al*.^36^ and the water molecules were modeled using the same O/H ReaxFF parameters as employed in previous ReaxFF descriptions^36–38^. Through validation, the ReaxFF reactive force field method was shown to provide an accurate account of the chemical and mechanical behavior of hydrocarbons^35^, graphene^39^, carbon nanotubes^40,41^ and other carbon nanostructures^38,42^. The force field parameterization is described in detail in Rahaman *et al*.^43,44^. All MD simulations of quasi-2D ice were performed by considering graphene sheets equilibrated in the *NP*_*xy*_*T* (constant number of atoms (*N*), constant pressure (*P*) in the direction parallel to the graphene sheets, and constant temperature (*T*)) ensemble at a pressure of 1 atm, with a time step of 0.10 fs using the Nose-Hoover thermostat and a coupling time constant of 10 fs to control the temperature of the entire system. During the simulations, the dimensions of the system remained constant, effectively melting the water at a constant volume. We carry out MD simulations for 50–100 ps, depending on temperature and density (*ρ*) of nanoconfined water. The positions of the C atoms of the walls are not fixed during the MD simulations.

## Results and Discussion

### Two-dimensional ice in graphene nanocapillaries

**Figure 1:**
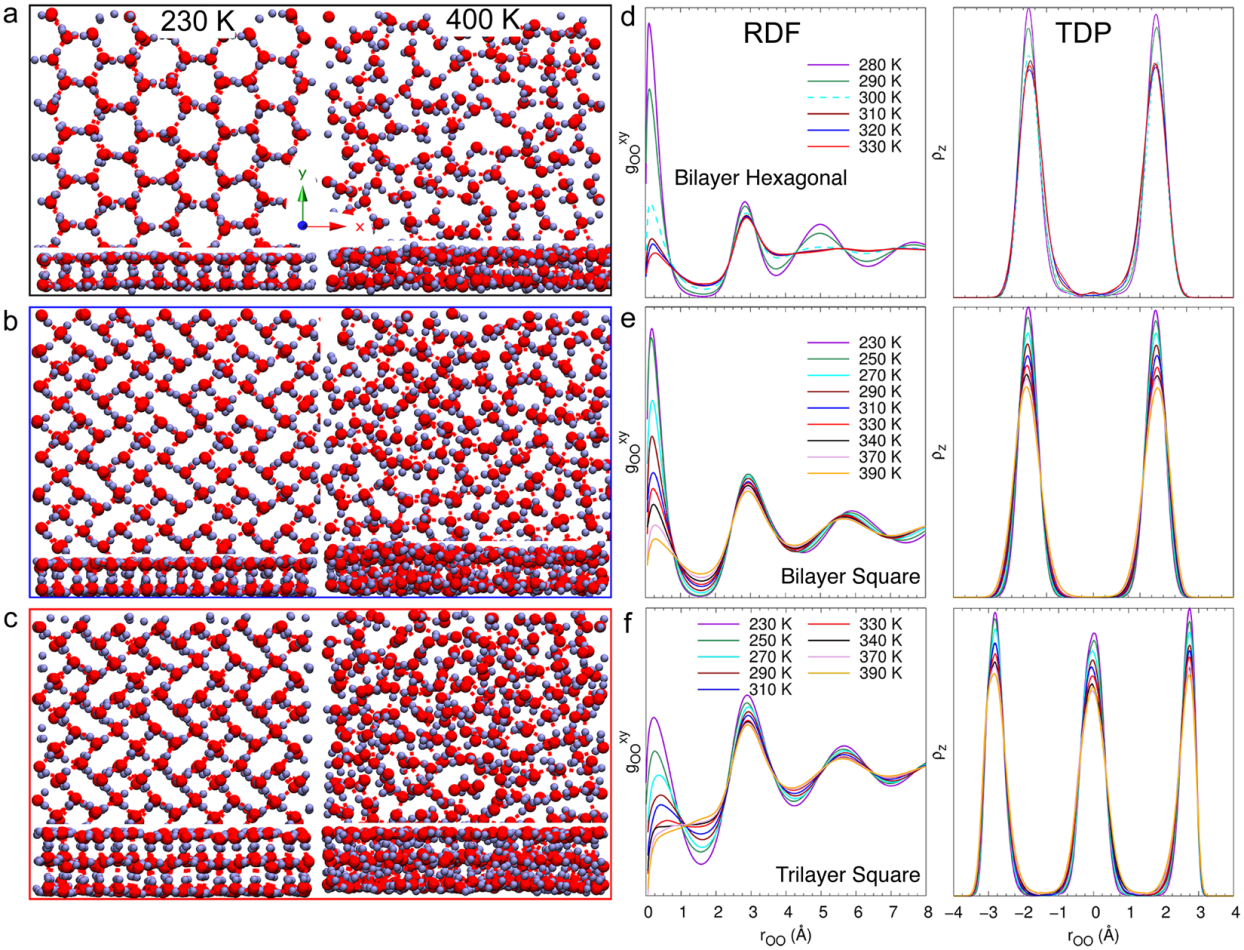
Nanoconfined 2D Melting. Different 2D structures in bilayer hexagonal and square ice and trilayer square ice. Snapshots of the top and side views of **(a)** bilayer hexagonal ice, **(b)** bilayer square ice and **(c)** trilayer square ice at 230 K (left) and 400 K (right). **(d**–**f)** Lateral RDF ${g}_{{\rm{OO}}}^{xy}$ (left) and TDP *ρ*_*z*_ (right).

We investigate the structure of 2D ice in nanocapillary confinement by choosing separations of 5.5 Å, 8.5 Å and 11.5 Å in order to accommodate one, two and three layers of water molecules, respectively. We used a periodic (32 × 48) supercell for the parallel graphene sheets with dimensions 139.2 × 120.56 Å^2^. We observe that on 2D nanoconfinement between graphene sheets, water forms ordered hexagonal phases at low densities and ordered square phases at high densities. The density of confined water is evaluated with respect to the entire pore volume without subtracting the vacuum gap between the graphene sheets and the water layer. In all future discussions, the computed water density is essentially the confinement density of water in the nanocapillary. The initial structures were obtained from grand canonical Monte Carlo (GCMC) simulations^45^, which were performed at a constant external chemical potential of liquid water (see Supplementary Material). Figure 1a–c shows the hexagonal and square ice structures obtained from MD simulations. We can immediately note that multilayer ice exhibits AA stacking. Energy minimized AA ice structures were found to be stable by 0.84 kcal/mol per water molecule as compared to ice structures with AB stacking. As shown in Fig. 1b and c our simulations predict an AA stacking for bilayer and trilayer square ice, which is also seen experimentally^1^. Bilayer and trilayer ice exhibit the same lattice constant as monolayered ice with *a* = 2.86 ± 0.02 Å, in good agreement with experiments^1^ (2.83 ± 0.03 Å). In hexagonal and square ice, every water molecule serves as a double donor and a double acceptor of hydrogen bonds and thus every water molecule is hydrogen-bonded to exactly four nearest-neighbor molecules. The different layers are held together by interlayer hydrogen bonding. The monolayer square ice structure is slightly rhomboidal, which is also seen from recent ab initio studies^46,47^; however, here we refer to it as square ice to follow previous convention^1^.

We observe that the layered square ice structure is stable only up to three layers and on subsequent addition of water layers the structure collapses. To investigate the stability of the layered ice structure, we examine the strength of the hydrogen-bond network between different layers. We consider a monolayer of square ice that fills the square lattice with *m* planar hydrogen bonds per layer. Our simulations show that for square ice, consisting of *n* layers with *n* ≥ 2, $\frac{n\times m}{3\times (n-1)}$ planar hydrogen bonds reorient to form interlayer hydrogen bonds connecting the layers. For bilayer ice, this rearrangement results in a hydrogen bond network with each of the layers containing 2*m*/3 planar hydrogen bonds and 2*m*/3 hydrogen bonds connecting the two layers. In trilayer square ice, all the layers have 2*m*/3 planar hydrogen bonds with *m*/2 hydrogen bonds connecting the layers, resulting in weaker interlayer hydrogen bonding between the layers. Subsequent addition of a water layer (*n* = 4) reduces the fraction of interlayer hydrogen bonds to *m*/3 between the interior layers, resulting in an unstable hydrogen-bonded lattice for multilayer ice with more than three layers of water. As a result, the layered square ice structure is stable for only up to three layers, which is also seen experimentally^1^. The ReaxFF MD calculations predict the structure of mono-, bi- and trilayer square ice in agreement with recent experiments^1^ and correctly reproduce the AA stacking observed in multilayer ice^1^.

**Figure 2:**
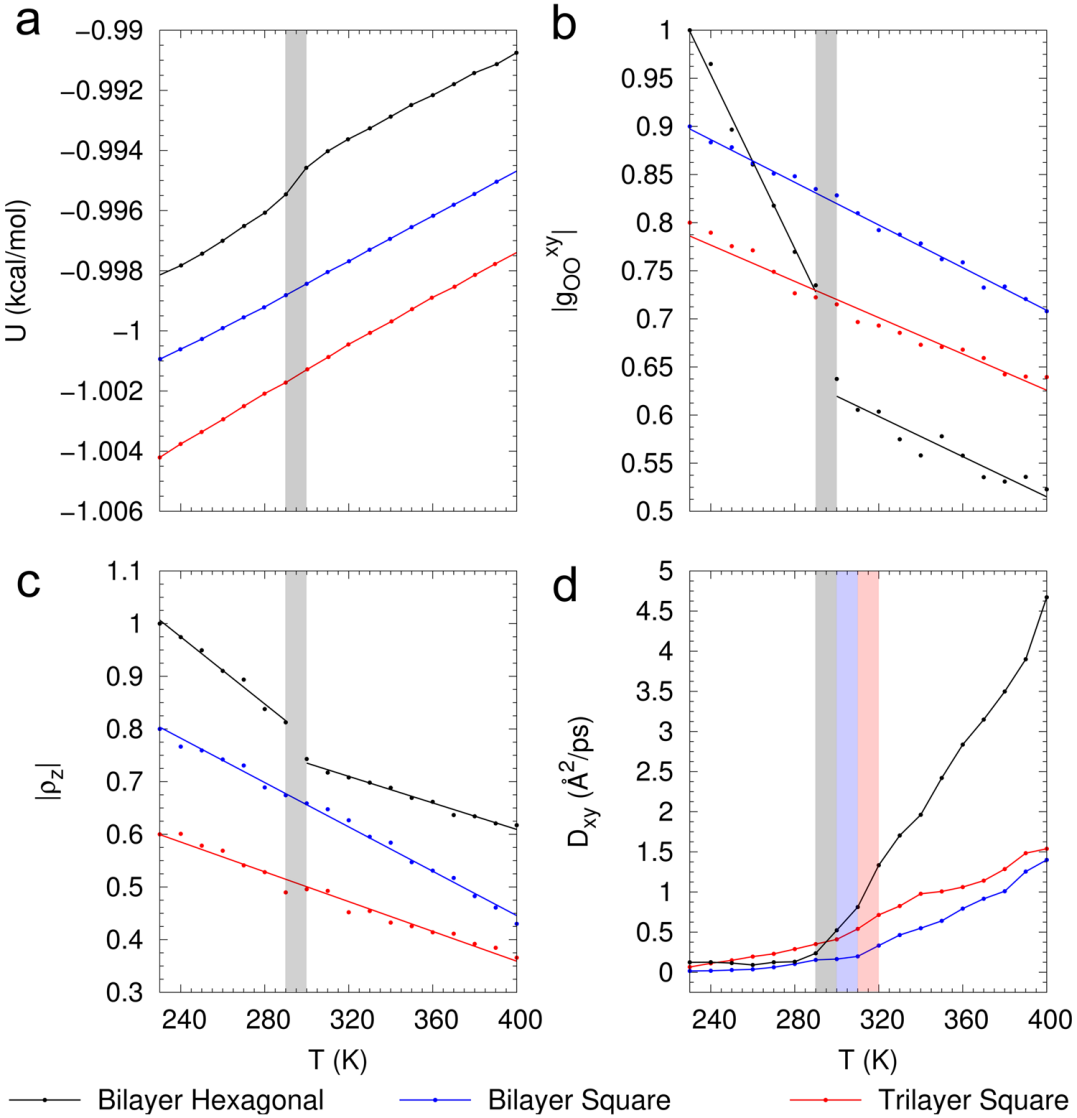
Nanoconfined 2D Melting. (**a**) Discontinuous versus continuous melting. Potential energy *U* as a function of temperature for bilayer hexagonal (0.97 g/cm^3^), bilayer square (1.34 g/cm^3^) and trilayer square ice (1.33 g/cm^3^). For bilayer hexagonal ice, on heating from 230 K, there is a sudden variation in *U* across the temperature range between 290 K and 300 K, characteristic of a first-order phase transition between solid and liquid. For bilayer and trilayer square ice, however, there is no sharp change in *U*, and *U* increases continuously as *T* is increased to 400 K. (**b**,**c**) Magnitude of first lateral peaks in RDF ${g}_{OO}^{xy}$ and TDP *ρ*_*z*_. The first peak of the RDF for bilayer hexagonal ice exhibits an abrupt jump as *T* is varied across 290 K and 300 K. Whereas the first peak of the RDF for bilayer and trilayer square ice varies continuously across the entire temperature range. The behavior of the TDP peak is identical to that of RDF. (**d**) Diffusion constant *D*_*xy*_. For both hexagonal and square ice we observe a slow diffusive regime at low temperatures, and a fast diffusive regime at high temperatures, characteristic of a solid to liquid transition. For hexagonal ice, the transition occurs over the temperature range 290 K to 300 K as also observed in *U* and RDF. For bilayer square ice, the transition from slow to fast diffusive regimes occurs over the temperature range 310 K to 320 K and for trilayer square ice, the transition occurs over the temperature range 300 K to 310 K.

We proceed to investigate phase transitions in ordered hexagonal and square ice structures, which is representative of confined 2D water at low and high densities, respectively. Figure 2a shows the potential energy *U* as a function of temperature for bilayer hexagonal, bilayer square and trilayer square ice. For bilayer hexagonal ice, we find a discontinuity in *U* in the temperature range between 290 K and 300 K, consistent with the presence of a first-order phase transition. In contrast, for bilayer and trilayer square ice we find that *U* varies continuously as the temperature increases from *T* = 230 K to 400 K. There are three possible explanations for the continuous change in *U*: (i) square ice remains in solid state from 230 K to 400 K; (ii) the presence of a weak first-order phase transition where the abrupt change in *U* is too small to be observed in our simulations and (iii) the presence of a continuous phase transition from solid to liquid state in square ice. The last two cases suggest the existence of a critical density above which the solid-liquid phase transition in square ice becomes (quasi-) continuous.

To determine which hypothesis is correct, we investigate various structural and dynamical properties of hexagonal and square ice as a function of temperature. We compute the lateral oxygen-oxygen radial distribution function (RDF) ${g}_{OO}^{xy}$ and the transverse density profile (TDP) *ρ*_*z*_ of oxygen between the graphene sheets to characterize the structural properties of hexagonal and square ice as a function of temperature. Figure 2b shows the variation of the magnitude of the first peak of the RDF with temperature. Hexagonal ice exhibits a sudden jump and change of slope in the peak magnitude of RDF over the temperature range between 290 K and 300 K, as also observed in *U*. This indicates a sudden structural change with increase in temperature, consistent with the presence of a first-order phase transition. We can observe that bilayer and trilayer square ice exhibits a continuous decrease in magnitude of the RDF peak, indicating that the structure changes continuously while melting. Figure 1d–f show RDFs for hexagonal and square ice at various temperatures across the solid-liquid phase transition. As shown in Fig. 1d, for hexagonal ice the RDF exhibits characteristic solid and liquid features at low and high temperatures, respectively. Up to temperatures of 290 K, the RDF exhibits long-range order that is typical of solids. In contrast, at 300 K the RDF shows typical liquid characteristics with a prominent first peak at *r*_OO_ = 2.86 Å, followed by rapidly decaying peaks indicating the disordered liquid state. This abrupt change in RDF as the temperature is varied from 290 K to 300 K for hexagonal ice is characteristic of a first-order phase transition as is also indicated by the sudden variation in *U*. In contrast, the RDF of bi- and trilayer square ice shows a gradual variation from solid to liquid behavior with increasing temperature, suggesting a continuous phase change (Fig. 1e,f). Recent MD studies by Jiao *et al*.^48^ report a similar behavior for monolayer square ice confined in graphene channels, where the 2D structure factor exhibits a continuous change on melting, over temperatures ranging from 310 K to 325 K. A similar behavior can also be observed from the TDP as shown in Fig. 1. Variations of the magnitude of the first peak of the TDP with temperature are shown in Fig. 2c. Identical to the behavior in RDF, hexagonal ice exhibits a sudden jump and change of slope in the magnitude of the TDP peak over the temperature range between 290 K and 300 K. In contrast, bilayer and trilayer square ice exhibits a continuous decrease in magnitude of the TDP peak, indicating a continuous structural change in the direction perpendicular to the confining surfaces while melting. TDPs for hexagonal and square ice across the solid-liquid phase transition are shown in Fig. 1d–f. The TDP of hexagonal ice exhibits an abrupt jump as the temperature increases from 290 K to 300 K, whereas the TDP of bilayer and trilayer square ice changes gradually with increasing temperature. In the high-density limit, as observed in the TDP, layering effects arising from confinement hinder the water molecules from leaving the layer and allow nanoconfined water to form relatively ordered structures as compared to typical bulk liquid water. Layering in nanoconfined water arises from confining boundary conditions and disappears as the density decreases or separation between the plates is increased^49–51^. The behaviors of RDF and TDP support the existence of two different phase transition regimes at low and high densities for 2D nanoconfined water, suggesting the existence of a critical density at which the nature of the phase transition changes.

At high densities, the potential energy as well as structural properties (RDF and TDP) exhibit continuous phase changes without a well-defined transition temperature between the solid-(like) and liquid-(like) phases. We proceed to characterize the dynamical properties of ice by computing the lateral diffusion constant *D*_*xy*_ from the lateral mean-square displacement (MSD) as a function of temperature (Fig. 2d). This density-dependent diffusivity for confined water has been reported by Jiao *et al*.^48^ for monolayer nanoconfined water in graphene channels. The diffusion constant for bilayer square ice shows two distinct regimes with an inflection temperature between 310 K and 320 K. From 230 K to 310 K, we observe a slow diffusive regime typical of solids, in which the diffusion constant linearly increases with temperature. At elevated temperatures between *T* = 320 K to 400 K, we observe a faster diffusive regime with diffusion constant increasing quadratically with temperature. The crossover point in the diffusivity can be considered as the transition temperature for the solid to liquid-like phase for square ice. For trilayer square ice this inflection temperature is observed at a reduced temperature between 300 K and 310 K. As was shown previously, trilayer ice has weaker interlayer hydrogen bonding as compared to bilayer square ice, which results in a lower inflection temperature. The existence of two dynamical regimes suggests that in the *ρ*-*T* phase diagram for 2D nanoconfined water, the high-density regime is actually divided into two regions that, although not connected by a first-order phase transition, can be identified by different dynamical regimes: solid-like and liquid-like.This can be likened to the pseudo-critical region above the liquid-gas critical point that is divided into two different regimes by the Widom line^52,53^: gas-like and liquid-like, distinguished by maxima in thermodynamic response functions or crossover in transport properties. Our simulations suggest that in the *ρ*-*T* phase diagram for 2D nanoconfined water, the first-order solid-liquid phase transition line therefore connects with a solid-liquid continuous transition line that is identifiable by a crossover in transport properties (see Fig. 5a). MD simulations with the TIP5P model^50^ for bilayer water molecules confined between two unstructured and smooth hydrophobic plates by Han *et al*.^25^ predict the existence of a similar connection point at which the first-order and continuous transition lines meet in confined water nanofilms. It is of interest to examine whether this continuous transition region terminates at a second critical point connecting to a single-phase region. Square ice is the densest ordered ice structure observed in 2D confinement from our GCMC simulations and denser ordered ice structures are not achievable. However, single-walled carbon nanotubes allow for denser packing of water molecules and are comparable model systems to investigate the *ρ*-*T* phase diagram for nanoconfined water in 1D confinement. The structure of ice in 1D confinement is investigated next.

### One-dimensional ice in carbon nanotubes

**Figure 3:**
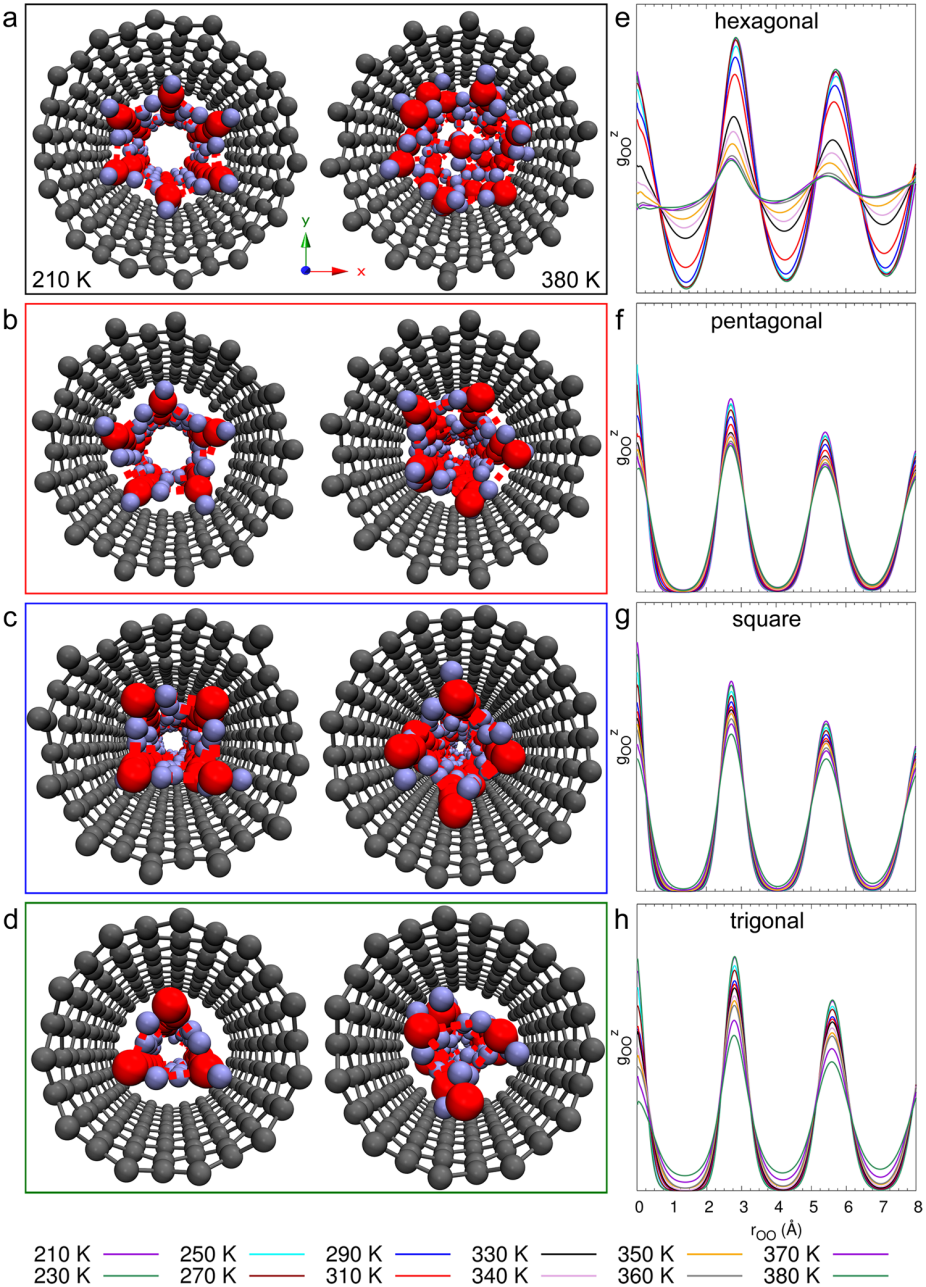
Nanoconfined 1D Melting. Different 1D ice structures in zigzag SWCNTs. Snapshots of the top view of **(a)** hexagonal ice in (14,0) **(b)** pentagonal ice in (13,0) **(c)** square ice in (12,0) and **(d)** trigonal ice in (11,0) zigzag SWCNTs at 210 K (left) and 380 K (right). (**e**–**h**) Axial RDF ${g}_{{\rm{OO}}}^{z}$ at different temperatures.

To examine 1D confinement of water in SWCNTs, we carry out MD simulations of water molecules confined within armchair (*R*,*R*) and zigzag (*R*,0) single-walled carbon nanotubes. We used armchair nanotubes with indices *R* = {6, 7, 8, 9}, corresponding to tubes with diameters of {8.1, 9.5, 10.8, 12.2} Å, respectively, and zigzag nanotubes with indices *R* = {11, 12, 13, 14, 15}, corresponding to tubes with diameters of {8.6, 9.4, 10.1, 10.9, 11.7} Å, respectively. The initial structures were obtained from GCMC simulations^45^, performed at a constant external chemical potential of liquid water as described in the Supplementary Material. Figure 3a–d shows the equilibrated *n*-gonal ice nanotubes composed of orderly stacked *n*-membered water rings, *n* = 2 (digonal) in (10,0), *n* = 3 (trigonal) in (11,0) and (6,6), *n* = 4 (tetragonal) in (12,0) and (7,7), *n* = 5 (pentagonal) in (13,0) and *n* = 6 (hexagonal) in (14,0) and (8,8) SWCNTs obtained from MD simulations. At larger diameters in (15,0) and (9,9) SWCNTs, the interior of the heptagon can accommodate additional water molecules and we obtain filled ice nanotubes as is also observed experimentally in neutron studies^27^. The one-dimensional lattice constant of the *n*-gonal ice nanotubes (*n* = 3–6) observed in our MD simulations, 2.86 ± 0.02 Å, is in good agreement with the periodicity reported in XRD experiments^32^, 2.8–2.9 Å. As in 2D ice, the density of 1D ice nanotubes are evaluated with respect to the entire pore volume without subtracting the vacuum gap between the SWCNTs and the water layer. The *n*-gonal ice nanotubes can be considered as rolled-up 2D monolayer square ice with roll-up vectors (*n*,0), resulting in denser packing of water molecules by elimination of one of the vacuum gaps between 2D square ice and graphene. The ordered *n*-gonal (*n* = 3–6) ice nanotubes cover a wide range of densities between 1.02 g/cm^3^ and 1.65 g/cm^3^ and we examine their phase behavior for temperatures from 210 K up to 380 K.

**Figure 4:**
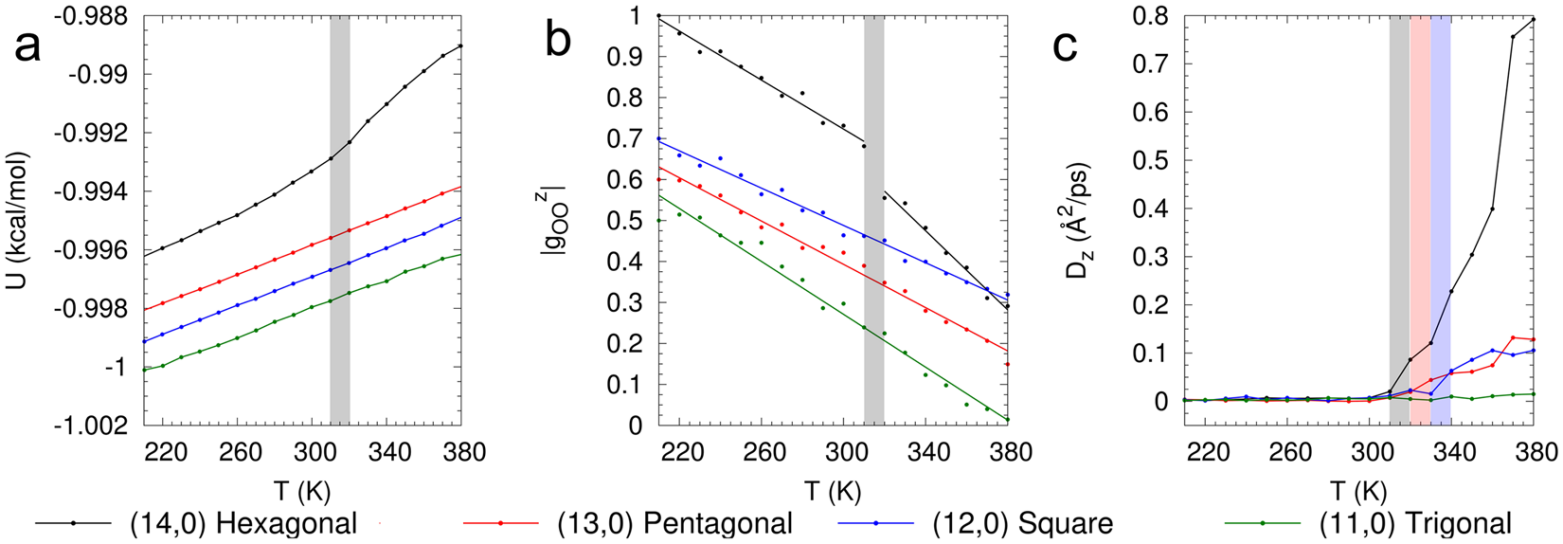
Nanoconfined 1D Melting. (**a**) Discontinuous versus continuous melting. Potential energy *U* as a function of temperature for hexagonal ice (1.07 g/cm^3^) in (14,0), pentagonal ice (1.23 g/cm^3^) in (13,0), square ice (1.44 g/cm^3^) in (12,0) and trigonal ice (1.53 g/cm^3^) in (11,0) zigzag SWCNTs. For hexagonal ice, on heating from 210 K, there is a discontinuity in *U*, which is characteristic of a first-order phase transition between solid and liquid. For pentagonal, square and trigonal ice, however, *U* increases continuously as *T* is increased to 380 K. (**b**) Magnitude of first peak of the axial RDF ${g}_{{\rm{OO}}}^{z}$ at different temperatures. The first peak of axial RDF for hexagonal ice exhibits an abrupt change as *T* is varied from 310 K to 320 K. The first peak of the RDF for pentagonal, square and trigonal ice decreases continuously across the entire temperature range from 210 K to 380 K. (**c**) Diffusion constant *D*_*z*_ for hexagonal, pentagonal, square and trigonal ice at various temperatures. For hexagonal, pentagonal and square ice we observe a slow diffusive regime at low temperatures, and a fast diffusive regime at high temperatures. However, for trigonal ice, we do not have two diffusive regimes. At these high densities we cannot differentiate the solid and liquid-like regimes based on mobility, which suggests a supercritical phase transition.

Figure 4a shows the variation of the internal energy *U* with temperature for various *n*-gonal (*n* = 2–6) ice nanotubes in zigzag SWCNTs. In the wide (14,0) SWCNT, containing the hexagonal ice nanotube, the potential energy exhibits a discontinuity in the temperature range between 310 K and 320 K. This is consistent with a first-order phase transition. The pentagonal ice nanotube in (13,0), square ice nanotube in (12,0) and trigonal ice nanotube in (11,0) do not exhibit this sharp change in *U* and instead, *U* increases continuously with temperature from 210 K to 380 K. The *n*-gonal ice nanotubes in armchair CNTs exhibit a similar behavior for *U*; see Supplementary Material). As in the case of 2D confined hexagonal and square ice, we investigate structural (RDF) and dynamic (diffusion constant) properties of 1D-confined ice to investigate the nature of these continuous phase transitions.

We proceed by computing the axial oxygen-oxygen RDF of ice nanotubes as a function of temperature to characterize their structural properties. Figure 4b shows the variation of the magnitude of the first peak of the RDF with temperature for various *n*-gonal (*n* = 2–6) ice nanotubes. We can observe that the hexagonal ice nanotube exhibits a sudden jump and change of slope in the magnitude of the RDF peaks in the temperature range between 310 K and 320 K, indicative of a first-order phase transition. Pentagonal, square and trigonal ice nanotubes exhibit a continuous decrease in the magnitude of the RDF peak with increasing temperature. The behavior of the RDF peak is consistent with the behavior of *U* and indicates a continuous structural change in pentagonal, square and trigonal ice across the solid-liquid phase transition. The RDF at various temperatures is illustrated in Fig. 3e–h. As shown in Fig. 3e, for hexagonal ice, the RDF exhibits an abrupt jump from solid to liquid behavior with increasing temperature, consistent with a first-order phase transition. In contrast, we can observe in Fig. 3f–h that the RDFs of pentagonal, square and trigonal ice exhibit a gradual change from solid to liquid behavior with increase in temperature. This gradual change in the RDF indicates a continuous phase transition from a solid to a liquid-like phase. At higher densities, layering effects that arise from confinement prevent the water molecules from leaving the layers and hence nanoconfined water has a relatively ordered structure compared to bulk liquid water. The behavior of the RDF indicates the existence of two different types of phase transitions at low and high densities in 1D nanoconfined ice. At high densities, the continuous change in the structural properties does not allow for identifying a transition temperature between solid and liquid-like phases.

Figure 4c shows the axial diffusion constant *D*_*z*_ for the *n*-gonal with *n* = 3–6 ice nanotubes as a function of temperature. For the pentagonal and square ice, we observe two distinct diffusive regimes with increasing temperature: a slow diffusive regime typical of solids at lower temperatures and a faster diffusive regime that is typical of liquids at higher temperatures. The resulting inflection point can be considered as the transition temperature for the solid to liquid-like phase at high densities. Interestingly, at even higher densities for the trigonal ice, we do not observe two distinctive diffusive regimes. The RDF in Fig. 3h and snapshot at 380 K (Fig. 3d) for trigonal ice indicate that the ice has transitioned to a disordered liquid-like state at higher temperatures. However, because of the high densities, the diffusivity remains low even after melting at higher temperatures. This suggests that in the *ρ*-*T* phase diagram for 1D nanoconfined water, there exists three regimes along the solid-liquid phase transition line: (i) a sub-critical solid-liquid transition regime at low densities (hexagonal ice) in which a first-order phase transition separates the solid and liquid phases, (ii) a continuous transition regime at intermediate densities (pentagonal and square ice) in which a continuous transition line characterized by a crossover in diffusivity separates the solid and liquid-like regimes and (iii) a single phase region (trigonal and digonal ice) at high densities where thermodynamic, structural and transport properties cannot distinguish the solid and liquid-like phases. The different phase-transition regimes on the *ρ*-*T* phase diagram can be considered as arising from the different confinement regimes under which the phase transition takes place.

**Figure 5:**
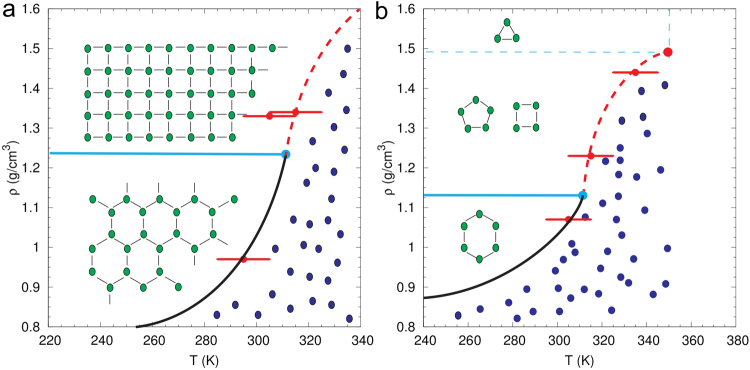
Schematic phase diagram in the density-temperature (*ρ*-*T*) plane for (**a**) 2D nanoconfined and (**b**) 1D nanoconfined water, evaluated from behaviors of *U*, RDF, TDP and diffusion constant. (**a**) In 2D nanoconfinement, at low densities water undergoes a phase transition between solid (green) and liquid (blue) phases by crossing a first-order (black solid) phase transition line. In contrast, at higher densities the phase change is continuous and the first-order line extends to a continuous transition line (red dashed) that is connected by a transition point (light blue circle); a critical point is not observed. (**b**) In 1D nanoconfinement, the first-order phase transition line (black solid) at low densities extends to a continuous phase transition line (red dashed) at intermediate densities. The two lines are connected by a transition point (light blue circle). The continuous transition line terminates at a critical point (red circle) that extends to a single-phase region. The ice phases have different symmetries and are separated by a phase boundary that is qualitatively shown by the light blue line.

Figure 5 shows the schematic phase diagrams for nanoconfined 2D and 1D water, evaluated from the behaviors of potential energy *U*, RDF, TDP and diffusion constant. In 1D confinement there exists two critical points: (i) a critical point connecting the first-order transition line with the solid-liquid continuous transition line and (ii) a second critical point connecting the solid-liquid continuous transition line to the single-phase region. Previous MD simulations of quasi-1D water in carbon nanotubes^3,20^ explore the phase behavior of nanoconfined water in the *P*-*T* state-space and predict the existence of a solid-liquid critical point at pressures of the order of 10^2^ MPa. The previous MD studies^3,20^ consider SWCNTs with diameters ranging from 11.1 Å to 14.2 Å containing *n*-gonal with *n* = 4–6 ice nanotubes. We extend our simulations to denser trigonal (*n* = 3) ice nanotubes and expand our understanding of the phase behavior of nanoconfined water by identifying different phase transition regimes characterized by the behavior of thermodynamic, structural and dynamic properties along the solid-liquid phase transition line. Recent studies report the existence of similar regimes along the liquid-vapor phase transition line, characterized by the Widom line^52,53^ extending from the liquid-vapor critical point. Equally exciting is the recent experimental evidence for the Widom line in unconfined water and, hence, of a second-order liquid-liquid critical point in the supercooled region of the water phase diagram by Kim *et al*.^54^.

In view of the fact that MD simulations^50^ of nanoconfined water between unstructured and smooth hydrophobic plates yield results that are qualitatively similar to our MD simulations employing structured graphene sheets, we expect that smooth hydrophobic cylindrical pores will provide qualitatively similar features as simulations on CNTs. Furthermore, MD simulations of Lennard-Jones fluids in cylindrical pores by Das *et al*.^55^ report a vanishing of the hysteresis loop during thermal cycling on reducing the diameter of the cylindrical pore, indicating transition to a continuous melting regime. Hence, we expect the features of the phase diagram reported here to be dimensionality specific and general to fluids confined in cylindrical pores and not limited to just water in CNTs.

**Figure 6:**
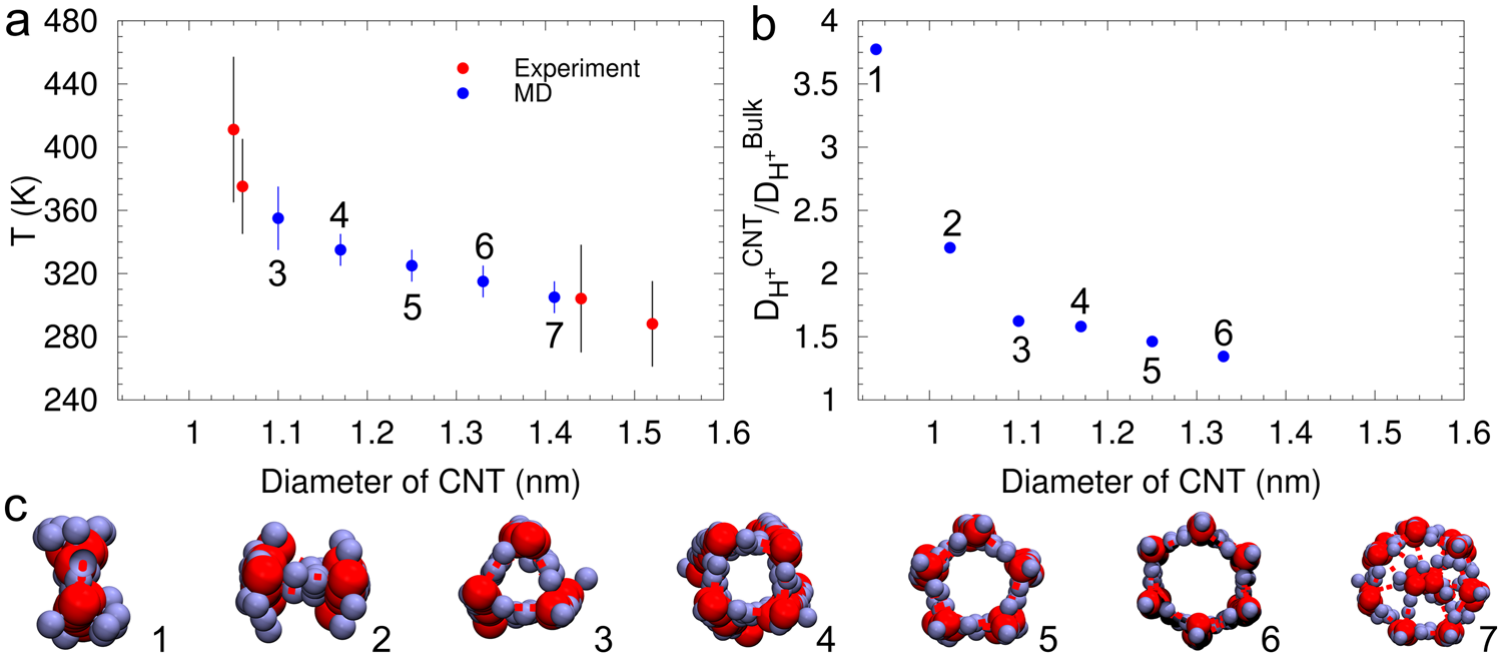
Phase transition temperatures and proton diffusion constants in 1D-ice. (**a**) Solid-liquid phase transition temperatures for 1D ice as a function of CNT diameter, obtained from experiments^2^ and MD simulations. (**b**) Proton diffusion constants in 1D ice normalized by the proton diffusion constant in bulk water as a function of CNT diameter. (**c**) Ice nanotubes. The various *n*-gonal ice nanotubes considered for the evaluation of the phase transition temperature and proton diffusion constants. The labels on the data indicate the corresponding *n*-gonal ice nanotube.

Raman spectroscopy experiments^2^ demonstrate that the solid-liquid phase transitions of confined water in CNTs are extremely diameter dependent, and freezing transitions at temperatures as high as 411 K for 1.05 nm SWCNTs were observed. Figure 6a shows that the freezing transition temperatures reported as a function of SWCNT diameter, obtained from MD simulations in this study are in good agreement with experimental data^2^ on water confined in single isolated chemical vapor deposition (CVD)-grown SWCNTs. We can observe from Fig. 6a that the freezing point exhibits extreme sensitivity to confinement length. Previous XRD^32,56^ and NMR^57^ experiments on polydisperse powders and suspensions of CNTs report lower solid-liquid transition temperatures for water confined in CNTs. However, experiments on polydisperse CNTs greatly reduce the resolution of the diameter-dependent behavior of confined water and this lower resolution can explain the lower transition temperatures reported in these experiments. We observe freezing point elevations ranging from 42 K to 82 K for the various *n*-gonal (*n* = 3–6) ice nanotubes, with freezing point elevation decreasing with increasing SWCNT diameter. The phase transition temperatures observed in our MD simulations are substantially higher than the values predicted by previous MD simulations^6,58^ and can be attributed to the different potentials employed in the MD simulations. These extreme phase transitions at temperatures above 273 K make hydrated SWCNTs viable candidates for latent heat storage systems^59^. Additionally, experiments^2^ report the existence of a continuous freezing transition in SWCNT of diameter 1.06 nm and first-order freezing transitions in larger tubes (1.15 nm–1.52 nm), consistent with our MD simulations. These advances are encouraging in regard to experimentally confirming the existence of a solid-liquid critical point.

### Proton diffusion in nanoconfined ice

At 300 K, 2D square ice is denser than liquid water and satisfies the ‘bulk ice rule’: every water molecule is hydrogen-bonded to exactly four nearest-neighbor molecules, whereas a water molecule in liquid water has a reduced average number of hydrogen bonds (between 3 and 4)^60,61^. Hence, 2D square ice has an ordered denser network of hydrogen bonds compared to liquid water and, as seen experimentally^1^ and from our simulations, remains in a solid state even at temperatures above 300 K. It will therefore be of interest to investigate proton and hydroxyl diffusion through this denser hydrogen bond network. To this end, we perform five separate MD simulations at 300 K with a proton in the 2D bilayer square ice structure to evaluate the average proton diffusion constant. We then repeat the same procedure to compute the average hydroxyl diffusion constant. To generate the initial geometries, a hydroxyl group is removed from a water molecule in the ordered 2D bilayer square ice to introduce a proton. Similarly, a hydrogen atom is removed from a water molecule to introduce a hydroxyl group in 2D square ice. This corresponds to a proton/hydroxyl concentration of 0.22 M. The MD simulation setup is the same as described before. We observe faster proton and hydroxyl diffusion in 2D bilayer square ice: 14.7 × 10^−5^ cm^2^/s and 11.3 × 10^−5^ cm^2^/s, respectively, compared to their respective values in liquid water^62^: 9.3 × 10^−5^ cm^2^/s and 5.6 × 10^−5^ cm^2^/s. A recent study by Achtyl *et al*.^63^ reports that proton exchange through naturally occurring atomic defects in single layer graphene exhibits much higher selectivity towards protons and much less hydrogen, oxygen, and methanol crossover than engineered nanoporous membranes.

In 1D molecular confinement we observe three different hydrogen-bonding configurations: (i) *n*-gonal ice nanotubes (*n* = 3–6) in which each water molecule is hydrogen-bonded to four nearest-neighbor molecules (ii) digonal ice nanotube (*n* = 2) in which each water molecule is hydrogen bonded to three nearest-neighbor molecules and (iii) 1D water chains (*n* = 1) in which each water molecule is hydrogen bonded to two nearest-neighbor molecules. Snapshots of the hydrogen-bonding configurations in various carbon nanotubes is provided as Supporting Material. The *n*-gonal ice nanotubes with *n* = 3–6 have a similar hydrogen bonding configuration as 2D square ice with each water molecule serving as a double donor and a double acceptor of hydrogen bonds. We investigate proton diffusion in digonal ice nanotubes and 1D water chains, in which each water molecule forms a lower number of hydrogen bonds, 3 and 2, respectively. Since the number of hydrogen bonds per water molecule is lower, the individual hydrogen bonds will be stronger and can be expected to enhance the proton diffusion. The proton diffusion constant in digonal ice nanotube in (10,0) and 1D water chain in (9,0) CNTs were obtained using the same procedure employed for 2D square ice. The proton diffusion constant in diagonal ice nanotube is 20.5 × 10^−5^ cm^2^/s and in 1D water chains, the proton diffusion constant increases further to 35.1 × 10^−5^ cm^2^/s. This is in good agreement with the proton diffusion constant of 42.0 ± 9.1 × 10^−5^ cm^2^/s observed experimentally in 1D water chains in sub-1-nm diameter CNT porins by Tunuguntal *et al*.^7^. The proton diffusion constants in 1D ice normalized by the proton diffusion constant in bulk as a function of CNT diameter is shown in Fig. 6(b). The melting temperatures and proton diffusion constants observed from our simulations are in good agreement with recent experimental studies^2,7^, validating the force field employed in our studies. Our results reveal the critical role that molecular confinement plays in enhancing the rates of proton transport via the Grotthuss mechanism, with the more confined water structures exhibiting faster proton diffusion.

## Conclusions

In summary, we have presented results from MD simulations of water under 2D and 1D nanoconfinement using hydrophobic structured walls. We characterize the thermodynamic, structural and dynamic behavior of nanoconfined water across the solid-liquid phase transition and identify different phase transition regimes along the solid-liquid coexistence line on the *ρ*-*T* phase diagram. We observe that quasi-2D water can melt continuously or discontinuously depending on the confined water density. 2D hexagonal ice exhibits a change of slope in potential energy *U* and sharp variations in lateral oxygen-oxygen RDF across the melting transition. Conversely, 2D square ice exhibits a continuous change in *U* and RDF across the phase transition and, hence, thermodynamic and structural properties cannot identify a freezing/melting point. However, sharp jumps in water diffusivity enables distinction between the solid and liquid phases of 2D square ice. We observe a similar behavior in quasi-1D ice nanotubes: Hexagonal ice nanotubes exhibit discontinuous behavior in energy *U* and axial oxygen-oxygen RDF across the melting transition; pentagonal and square ice exhibits continuous changes in *U* and RDF across the melting transition. However, the solid and liquid phases can be distinguished based on the water diffusivity; Trigonal ice exhibits a continuous melting transition where thermodynamic, structural and transport properties cannot distinguish the solid and liquid-like phases. This suggests the existence of a solid-liquid critical point in quasi-1D nanoconfined water. The behavior of *U*, RDF and diffusion constant of nanoconfined water across the solid-liquid phase transition was employed to propose new *ρ*-*T* phase diagrams of quasi-2D and -1D nanoconfined water. We observe extreme elevations in the freezing point of water confined in SWCNTs in agreement with recent experiments by Agrawal *et al*.^2^. The freezing point exhibits a unique and extreme sensitivity to confinement length. Our results demonstrate a different behavior of nanoscale confined fluids from that in the bulk, indicating new directions for investigations of phase transitions of confined fluids. We observe ultrafast proton and hydroxyl transport in quasi-1D and -2D ice at 300 K, exceeding those of bulk water up to a factor of five, opening up interesting opportunities for applications in proton as well as anion exchange fuel cell membranes.

## Electronic supplementary material

Supplementary Material
