# Supplementary material for: Phase transitions of ordered ice in graphene nanocapillaries and carbon nanotubes
Source: Sci Rep. 2018 Mar 1;8:3851. doi: 10.1038/s41598-018-22201-3 (PMC5832794; doi:10.1038/s41598-018-22201-3)
Supplement: Supplementary file 1 — Supplementary Material [file 41598_2018_22201_MOESM1_ESM.pdf]

Supplementary Material: Phase transitions of ordered ice in graphene nanocapillaries and carbon nanotubes

Muralikrishna Raju[1], **Adri van Duin**[2], **and Matthias Ihme**[1,*]

[1]Department of Mechanical Engineering, Stanford University, Stanford, CA 94305
[2]Department of Mechanical and Nuclear Engineering, The Pennsylvania State University, University Park, PA 16802
[*]mihme@stanford.edu

1 Methods

We apply hybrid Grand Canonical Monte Carlo/Molecular Dynamics (GCMC/MD) simulations[1,2] based on the Metropolis algorithm to obtain a set of ground states of nanoconfined water in graphene nanocapillaries and single walled carbon nanotubes (SWCNTs). The possible MC moves include: (1) inserting a water molecule into the system at a random position, (2) removing a randomly selected water molecule from the system, or (3) translate a water molecule to a new random position in the system. Coordinates for H_2O insertion and H_2O translation steps were chosen randomly such that any unoccupied position in the simulation box could be selected. The chemical potential of water employed in our GCMC simulations is -260.2 kcal/mol, as determined by the Widom Insertion method. In our hybrid method, we introduce an energy minimization step after each MC trial move. In this study, the energy minimization step consists of a conjugate gradient (CG) geometry optimization, with a convergence criterion of 0.5 kcal/mol between subsequent CG steps. The GCMC temperature scans at constant chemical potential are combined with molecular dynamics runs of 12.5 ps in the NVT-ensemble for every 50 successful GCMC moves. The hybrid GCMC/MD method allows the movement of C and intercalated H_2O molecules during the energy minimization and MD runs and enables the system to explore the phase space at the simulation temperature. This enables the method to investigate the role of intercalated H_2O on subsequent intercalation. The development and implementation of the hybrid GCMC/MD method in ReaxFF is described in detail in Senftle *et al.*[1,2]

2 Supporting Information

References

1. Senftle, T. P., Meyer, R. J., Janik, M. J. & van Duin, A. C. T. Development of a ReaxFF potential for Pd/O and application to palladium oxide formation. *J. Chem. Phys.* **139**, 044109 (2013).

2. Senftle, T. P., van Duin, A. C. & Janik, M. J. Determining *in situ* phases of a nanoparticle catalyst via grand canonical monte carlo simulations with the ReaxFF potential. *Catal. Commun.* **52**, 72–77 (2014).

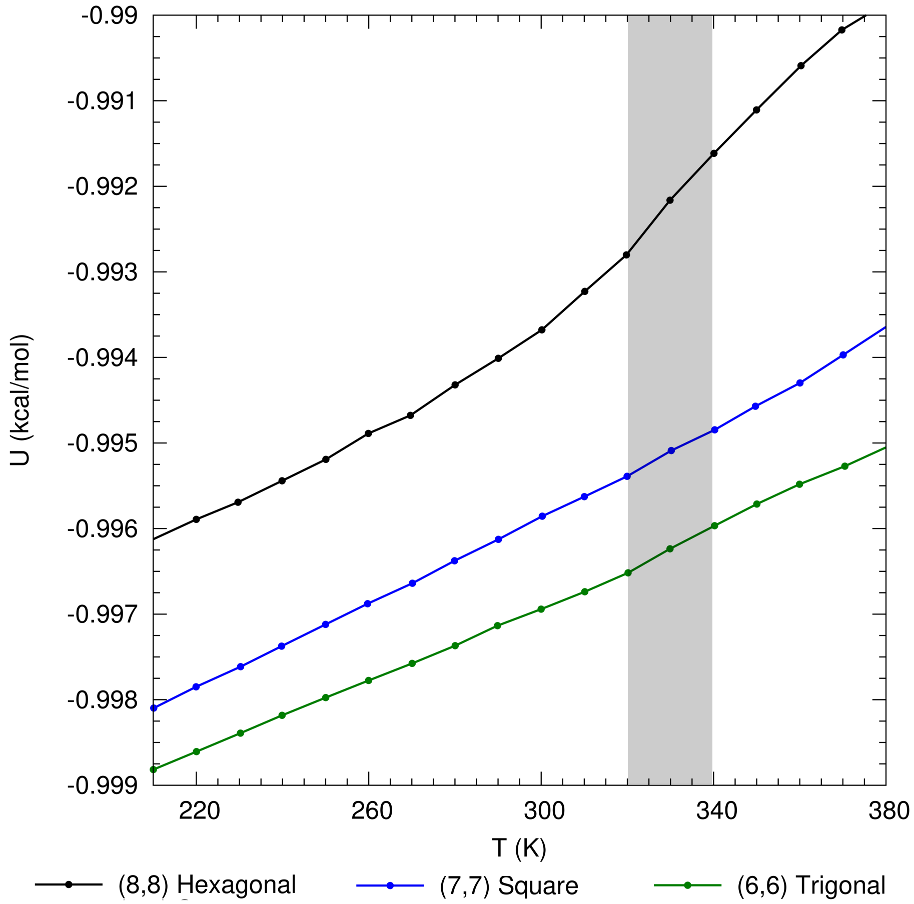

Figure 1. Discontinuous versus continuous melting. Potential energy U as a function of temperature for hexagonal ice (1.02 g/cm^3) in (8,8), square ice (1.32 g/cm^3) in (7,7) and trigonal ice (1.65 g/cm^3) in (6,6) armchair SWCNTs. For hexagonal ice, on heating from 210 K, there is a discontinuity in U, which is characteristic of a first-order phase transition between solid and liquid. For square and trigonal ice, however, U increases continuously as T is increased to 380 K. The behavior of various n-gonal ice in armchair SWCNTs mirrors their behavior in zigzag SWCNTs.

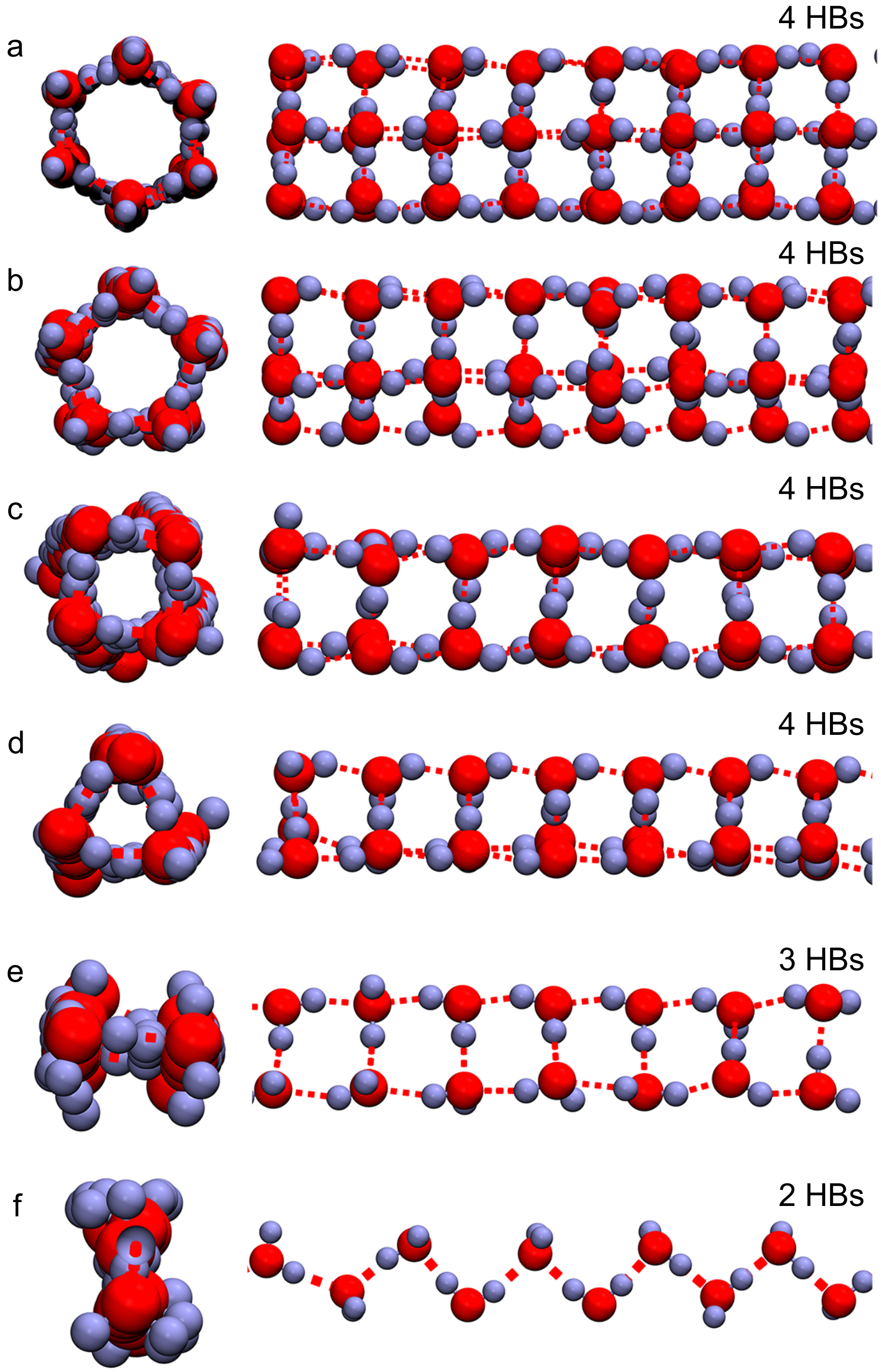

Figure 2. Hydrogen-bonding configurations in various *n*-gonal (n = 1-6) **ice nanotubes.** Snapshots of the top (left) and axial (right) view of **(a)** hexagonal, **(b)** pentagonal, **(c)** square, **(d)** trigonal, **(e)** digonal nanotubes and **(f)** 1D water wire. The number of hydrogen bonds (HBs) associated with a water molecule in the ice nanotubes is listed.
